# The Relationship between Nutritional Status and Functional Capacity: A Contribution Study in Institutionalised Portuguese Older Adults

**DOI:** 10.3390/ijerph18073789

**Published:** 2021-04-05

**Authors:** Catarina Caçador, Edite Teixeira-Lemos, Jorge Oliveira, João Pinheiro, Filipa Mascarenhas-Melo, Fernando Ramos

**Affiliations:** 1 Faculty of Pharmacy, University of Coimbra, 3000-548 Coimbra, Portugal; cacasabel@hotmail.com; 2 Agrarian Scholl-IPV and CERNAS-IPV Research Centre, Polytechnic Institute of Viseu, 3504-510 Viseu, Portugal; etlemos3@gmail.com (E.T.-L.); joliveira@esav.ipv.pt (J.O.); 3 Faculty of Medicine, University of Coimbra, 3000-548 Coimbra, Portugal; jpinheiro@fmed.uc.pt; 4 Department of Pharmaceutical Technology, Faculty of Pharmacy, University of Coimbra, 3000-548 Coimbra, Portugal; filipamelo99@yahoo.com; 5 REQUIMTE/LAQV, R. D. Manuel II, Apartado 55142, 4051-401 Porto, Portugal

**Keywords:** older adults, nursing homes, exercise, nutrition, cognition, Portugal

## Abstract

Demographic aging of the population allied with the new family structures and societal dynamics is generating an increasing demand for institutions for older adults. Nutritional status is a key health determinant that impacts the quality of life among older adults. Hence, the aim of the present study was to evaluate the relationship between nutritional status and nutritional risk, functional capacity, and cognition in institutionalised Portuguese older adults by a cross-sectional study in 15 institutions. Nutritional status (body mass index (BMI), waist circumference (WC), nutritional risk (mini nutritional assessment (MNA)), degree of functional independence (Barthel index (BI)), and cognitive ability (mini mental state examination (MMSE)) were assessed. Of the 214 older adults evaluated, 28.0% were at risk of malnutrition, 69.6% were mildly functional dependent, and 39.3% presented minor cognitive impairment. The risk of malnutrition increased functional dependence and cognitive impairment. The MNA score, but not the BMI or WC, was related to disability and deficits in cognition. A differential interdependence was found between nutritional, cognitive, and functional status. Strategies to improve self-care and well-being in nursing homes should consider a correct diet and a closer evaluation of nutritional risk to preserve cognition, independence, and autonomy.

## 1. Introduction

Aging is a global phenomenon that has a significant impact on population pyramids [1]. Increase in life expectancy, declining fertility, mortality rates, and reduced population growth are key factors that result in increasing numbers of older adults, thus leading to an increase in the proportion of adults over 65 years of age [2]. In Europe, older adults accounted for 15% of the total population in 1971, 20% in 2000, and it is expected to reach 35% in 2050 [3]. In Portugal, the scenario is similar. The prevalence of older people ranged from 8% in 1960 to 17% in 2007, and projections for 2050 suggest that the number will increase to 32% [1,4]. It is worth noting that population distribution is not homogeneous across countries. For instance, central Portugal, which includes the district of Viseu, presents one of the highest aging indexes in the country.

The demographic aging of the population, coupled with new family structures and societal dynamics, is generating an increasing demand for institutions for older adults [5]. The lack of family support or decent housing, insufficient economic resources, and the increasing insertion of women in the labour market are some of the factors that justify why families resort to institutionalisation [6]. The institutionalisation process is complex and involves radical changes in daily life routines [7]. As a consequence, a deterioration of the nutritional, cognitive, and functional status is observed.

Malnutrition is becoming an alarming phenomenon among institutionalised older adults and is oftentimes underdiagnosed. In the general population, this condition has a prevalence rate between 5–12%, while in institutionalised adults, the prevalence is as high as 52–85%, depending on the criteria and methodology used in each study [8]. In older persons, malnutrition increases morbidity and mortality, reduces the quality of life, and increases the length of hospital stay. Furthermore, malnutrition is associated with deteriorating functional status, weakening of the immune system, increased risk of infections, poor wound healing, and sarcopenia [2].

Continuous assessment of the nutritional status, functioning, and cognitive ability of institutionalised older adults is crucial to prevent their dramatic decline and improve the quality of life in this population [9]. Although functional and cognitive abilities and malnutrition are reported to be very closely linked to each other, the differential association between nutritional risk and nutritional status with the incidence of disability for basic activities of daily living and cognitive impairment remains limited. The present study aims to (1) characterise the nutritional and functional status of an institutionalised population in one of the most rapidly aging regions of Portugal, (2) examine the possible relationship between nutritional status (as evaluated by the BMI and WC) and nutritional risk (as measured by MNA), and (3) study whether nutritional status and nutritional risk could be associated with a decrease in functional outcomes for activities of daily living (as assessed by BI) and with cognitive impairment (as assessed by MMSE) in institutionalised older adults.

## 2. Materials and Methods

### 2.1. Subject Recruitment and Data Collection

A cross-sectional study was conducted using data collected from residents living in home-care residences throughout the district of Viseu (Portugal). The inclusion criteria were subjects (1) aged 65 or older, (2) living in nursing homes for at least the last three months, (3) presenting a BI ≥40 points, (4) able to walk (with or without walking aid), and (5) who accepted to participate in the study. The exclusion criteria were (1) staying temporarily or living in the institution for <3 months, (2) suffering from cognitive impairment, (3) presenting psychiatric disorders or dementia, and (4) refusing to participate in the survey. Data were collected in 15 institutions. All participants gave their written informed consent for inclusion before they participated in the study. The study was conducted in accordance with the guidelines of the World Medical Association’s Declaration of Helsinki (as revised in Brazil 2013), and the protocol was approved by the Ethics Committee of the Polytechnic Institute of Viseu (Ref. 01/sub/2021). Complete information on demographics, nutritional status, nutritional risk screening, functional and mental status, was routinely collected through self-report using standardised questionnaires administered by trained researchers. In demography, formal education was categorised according to the years of formal education and the cut-off points to MMSA: 0 years = illiterate, 1–11 years, >11 years.

### 2.2. Anthropometric Measurements and Nutritional Indicators

WC and BMI were used to assess nutritional status according to standard criteria. Anthropometric measurements, such as weight, height, and WC, were undertaken. Height (rounded to the nearest 0.1 cm) was measured using a measuring tape, with participants standing upright against a wall without shoes. WC was defined as the midpoint between the lower rib and the upper margin of the iliac crest and was measured to the nearest 0.1 cm. WC classifications were based on the cut-off values recommended by the National Cholesterol Education Program Adult Treatment Panel III (NCEP ATP III)—high risk of cardiovascular disease when WC ≥102 cm in males and ≥88 cm in females. When the older person had deformations of the spinal column, the sitting knee height was measured in accordance with Chumlea et al. [10]. BMI was calculated as weight (kg)/height (m)^2^ and classified according to Lipschitz [11]. Participants were weighed using a digital chair scale to the nearest 0.1 kg.

Nutritional risk was established by the MNA, which was validated for the Portuguese older adult population (≥65 years old) [12]. The MNA with a total score of 30 points consists of 18 questions, allowing several assessments: anthropometric assessment (BMI, weight loss, mid-upper arm circumference, and calf circumference), global assessment (mobility, prescription drugs, independent life, psychological stress, or acute disorder, pressure sores or decubitus ulcers and neuropsychological problems), dietary assessment (complete meals eaten daily, decline in food intake, fluid consumption, protein intake, fruit and vegetable intake and mode of feeding) and self-assessment (self-view of nutritional status and self-view of health status). The MNA classifies individuals into three categories—malnourished (<17 points), at risk of malnutrition (17–23.5 points), and normal nutritional status (≥24 points) [13].

### 2.3. Performance in Activities of Daily Living (ADL)

BI is an index used to evaluate the functional ability of the older adult in 10 ADL (ambulation, chair/bed transfers, bathing self, personal hygiene, stair climbing, feeding, toileting, bowel control, bladder control, dressing). The total score of BI ranges from 0 to 100 points and classifies the individuals’ level of dependence as follows: <20, totally dependent; 20–39, very dependent; 40–59, partially dependent; 60–79, minimally dependent; and 80–100, independent [14].

### 2.4. Cognitive Performance

Cognitive status was evaluated by the MMSE validated for the Portuguese population [15]. This is one of the most widely used instruments as a screening for cognitive impairment. It includes 30 items, which assesses temporal and spatial orientation, working memory, recall, attention, arithmetic capacity, linguistic, and visual–motor skills. The maximum score is 30 points (one point per correct item). The minimum cut-off value for adequate cognitive functioning is set accordingly to the level of education of the participant. In this study, cognitive impairment was defined using the following score cut-off points: ≤15 for illiterate, ≤22 for subjects with 1–11 years of education, and ≤27 for people >11 years of education [16].

### 2.5. Statistical Analysis

The collected data were analysed using IBM SPSS Statistics, version 26.0 (IBM SPSS Statistics for Windows, Version 23.0. Armonk, NY, USA). Data analysis included descriptive statistics such as mean, standard deviation, and frequencies. The independent variables t-test or the analysis of variance (ANOVA) with Bonferroni post hoc tests were used to compare the means of the continuous variables according to the MNA and BMI classes. The effects of gender and age (by classes) were performed using chi-square tests, considering counting variables. Pearson’s Correlation was used to evaluate correlations between continuous variables. The significance level of *p* < 0.05 was considered for all statistical analyses.

## 3. Results

### 3.1. General Characteristics of the Population

Table 1 summarises the demographic, nutritional, functional, and mental status of the study participants.

The mean age of the 214 homecare participants was 82.3 ± 6.1 years old, and 72% (n = 154) were female. Most of them presented a low educational level (illiterate or four years of schooling = 88.8%) and were widowed, separated, or divorced (74.3%). Women were more likely than men to be widowed and less likely to get married. On average, men had a higher WC than women (101.9 ± 10.0 vs. 94.6 ± 10.5), but there were no significant differences between men and women in the percentages of participants with WC above the risk cut-offs (102 cm for men and 99 cm for women) [17]. BMI values ranged from 16.0 kg/m^2^ to 43.3 kg/m^2^ with a median value of 28.3 ± 4.9 kg/m^2^. Based on Lipschitz classification [11], 7.5% of the subjects were underweight (BMI < 22 kg/m^2^), 34.6% were normal weight (BMI 22–27 kg/m^2^), and 57.9% were overweight (BMI > 27 kg/m^2^). The percentages of men and women in the three BMI categories did not differ significantly. Overall, 60 participants were at risk of malnutrition (RM) (28.0%), and 154 (72%) were well nourished (WN) with no significant gender differences. BI scores indicated that the majority of patients (69.6%) were minimally dependent on ADL. A total of 84 (39.3%) participants presented cognitive impairment through the MMSE screening (Table 1). Among them, women were more affected than men (*p* < 0.01).

### 3.2. Age Effect on Nutritional Status, Nutritional Risk, Self-Dependence in Activities of Daily Living and Cognitive Performance

In Table 2, MNA, BMI, WC, BI and MMSE are reported according to age classes. There were no differences according to age in any of the different variables.

### 3.3. Effect of Nutritional Status and Nutritional Risk on Functional and Cognitive Performance

The influence of nutritional status on nutritional risk, functional, and cognitive performance was evaluated. The results showed that MNA was statistically different between the classes of BMI (*p* < 0.001) and BI (*p* < 0.05); nevertheless, there were no differences when considering cognitive evaluation (Table 3).

### 3.4. Nutritional Risk

Malnutrition was not observed among the residents; however, 28% of the study population was found to be at risk of malnutrition (Table 1). The influence of MNA on the ability to perform ADL and on cognitive status was verified (Table 4).

These observed differences between the MNA scores were significant (*p* < 0.05). The results indicated that the improvement in nutritional status was accompanied by an increase in BMI and an improvement in BI and cognitive function. Thus, it is evident that the risk of malnutrition increases the dependence of residents.

The correlation analysis between nutritional status, nutritional risk, and functional and mental disability assessment (BI and MMSE) is shown in Table 5. There were no correlations between nutritional status (BMI and WC) and BI and MMSE. However, a slightly positive correlation was observed between nutritional status and nutritional risk (*p* < 0.001). MNA had a moderate positive correlation with BI and MMSE. Therefore, the ability to perform ADL and cognitive functions is directly related to a comprehensive nutritional assessment measured by the MNA in institutionalised older adults.

## 4. Discussion

The present study characterised a population of institutionalised Portuguese older adults and evaluated the association between their functional performance, cognitive ability, nutritional status, and nutritional risk.

The study population was mainly female with less than four years of schooling and mostly without a partner (single, widowed, separated, or divorced). These characteristics were observed in other Portuguese studies with institutionalised individuals [18,19]. This phenomenon may be related to excess male mortality and life expectancy, which is known to be higher in females [3]. Longer life expectancy often translates into more health problems that, in addition to physiological differences in brain structure and function, may explain gender variation in cognitive aging [20,21]. In fact, deficits in cognition were observed to be significantly different between males and females with a higher prevalence among women. Few studies mentioned significant differences in gender-based cognition, while others reported no difference [22,23]. In addition, this study showed a moderate vulnerable older adult population, with minimal dependence on ADL (69.6%) and impaired cognition (39.3%).

Age is a well-documented factor that contributes to the progressive decline in cognitive and functional abilities and makes individuals prone to nutritional deficiencies [2]. However, in this study, age was not considered a predictive factor for nutritional status or risk, self-dependence in ADL, and cognitive status. In fact, different authors have suggested that there are other risk factors more powerful than considering age. Comorbidities such as diabetes, hypertension, and obesity, and educational level are some examples [24]. Hence, since our sample constitutes 88.8% of low educational level (illiterate or 11th year of schooling or less) and have considered age with other comorbidities, there may be an additional effect of confusion associated with age.

Overweight or obese patients are often identified by BMI, which is an objective measure of body fat based on height and weight. High BMI is considered a risk factor for the health of the older person since it is associated with deterioration in the quality of life and high rates of morbidity and mortality [25]. BMI characterised 57.9% of individuals to be overweight. Although being overweight is associated with a decrease in physical well-being, the individuals studied did not show an impaired ability to perform normal activities of daily living since the BI values were similar to those with normal BMI [26]. The use of BMI as a surrogate for adiposity is controversial in older adults because (1) individuals lose height as they age, creating an overestimation of BMI values [27] and (2) BMI fail on identifying older adults with obesity, and it is unable to distinguish between peripheral and visceral obesity. Therefore, older adults with central obesity presenting a normal BMI may also be at risk for adverse cardiometabolic dysfunction [28,29]. Moreover, the continuous loss of muscle mass during the aging process when it is associated with obesity (sarcopenic obesity) can go unnoticed, suggesting that it is possible to have a high BMI and be inappropriately nourished [30]. These considerations lead to a reflection about the measures for weight management in institutionalised old adults. In the present study, the high incidence of independent overweight individuals suggests a medical/nutritional evaluation in order to manage the benefits vs. the risks of any future interventions. Low-weight seniors (7.5%) presented a significant decrease in physical abilities, while no differences in mental well-being were verified. These results might be assigned to a decrease in activity due to malnutrition or sarcopenia.

Anthropometric values (e.g., BMI and WC) were closely related to nutritional status, providing detailed information on the different components of the body structure, especially the muscular and fatty components [26]. A positive correlation was observed between BMI and WC and nutritional risk; however, statistical significance was only achieved between BMI and MNA score, despite this correlation not being strong. As expected, a strong correlation (r = 0.72) was verified between both anthropometric values. Although only 34.6% of the institutionalised older adult had normal BMI, the MNA assessment indicated that 72% of the population had a normal nutritional status. This discrepancy is explained by the multifactorial variables evaluated in MNA, in addition to anthropometric values, which provide a closer insight into whether the older adults are adequately nourished or not [13].

In comparison with other studies conducted in Europe, similar frequencies for risk of malnutrition were observed: 42% in Germany, 51% in Spain, 46% in Italy, compared to 42.7% in our study [31,32,33]. Thus, more research remains to be conducted on the institutionalised older adults to balance their nutritional status.

In line with the previous observations, the older adult studied at risk of malnutrition presented significantly increased cognitive impairment, compared to the well-nourished.

A slightly positive correlation was found between MNA scores and MMSE values (r = 0.3, *p* < 0.0001). Previous studies conducted in other institutions also demonstrated a correlation between nutritional status and cognitive function. Malnourished older adults or even at risk of malnutrition presented lower cognitive abilities than those with a normal nutritional status [34].

Similarly, different studies obtained an equivalent relationship between malnutrition and functional dependence [35]. Lower BI scores were found mainly in the poorly nourished older adult than in those with better nutritional status. Our research showed a positive correlation between MNA and BI (r = 0.4, *p* < 0.0001). In general, well-nourished individuals perform better in ADL.

Cognitive and functional abilities were also found to be interconnected (r = 0.35, *p* < 0.0001). Previously, a systematic review pointed to a strong but unilateral association between cognitive and functional impairment [7]. Deficits caused by cognitive decline can lead to disability, thereby reducing and/or losing the ability to perform activities of daily living. However, functional impairment does not result in the deterioration of cognitive functions.

Although statistical significance was observed between the variables, the correlation values were weak. However, these results do not invalidate the relationship between nutritional, cognitive, and functional status. On the contrary, these findings reinforce the need to monitor and intervene in each of these areas to enhance a balanced quality of life.

Studying this sample of older adults does not allow us to generalise the obtained results to the whole institutionalised Portuguese seniors. However, to the best of our knowledge, this study is the first to differentially evaluate nutritional, cognitive, and functional status in institutionalised Portuguese older adults. Nevertheless, the absence of random sampling and the sample size limits the representativity of the study. A larger random sample would possibly have strengthened correlation values between nutritional, cognitive, and functional abilities. Moreover, the results may be biased due to the refusal to participate in the study and the nature of the responses given in the questionnaire. Cognitive impairment may influence MNA responses since memory problems make it difficult for people to remember factors related to the type of ingested food or liquids and even the number of meals per day. In addition, the exclusion criteria could also withdraw the strength of the correlations since individuals with strong cognitive and functional impairments were not included in the study. However, there is no reason to believe that considering these weak points will drastically change the results.

## 5. Conclusions

This cross-sectional study shows a moderately vulnerable older adult population. Most of the older adults presented minimal functional dependence on ADL, normal nutritional status, and no cognitive impairment. A differential interdependence was found between nutritional, cognitive, and functional status. The risk of malnutrition is related to higher functional dependence and cognitive impairment. The need to implement cognitive and motor stimulation programs, and interventions aimed at enhancing a correct diet has been reinforced in order to preserve the nutritional, cognitive, and functional capabilities in institutionalised older adults. These programs should focus on achieving a greater level of independence and autonomy, thereby leading to an improvement in self-care and the overall well-being of these populations.

## Figures and Tables

**Table 1:** Population demographics, nutritional, functional, and cognitive characteristics by gender.

Variables	Total	Female	Male	*p*-Value
Educational Status				
Illiterate	79 (36.9)	60 (39.0)	19 (31.7)	0.549
1–11 years	124 (57.9)	87 (56.5)	37 (61.7)	
>11 years	11 (5.1)	7 (4.5)	4 (6.7)	
Civil Status				
Single	25 (11.7)	20 (13.0)	5 (8.8)	0.004
Married	30 (14.0)	14 (9.1)	16 (26.7)	
W/S/D	159 (74.3)	120 (77.9)	39 (65.0)	
WC				
Normal	132 (61.7)	100 (64.9)	32 (53.3)	0.080
High risk	82 (38.3)	54 (35.1)	28 (46.7)	
BMI				
Underweight	16 (7.5)	10 (6.6)	6 (10.0)	0.680
Normal	74 (34.6)	54 (35.1)	20 (33.3)	
Overweight	124 (57.9)	90 (58.4)	34 (56.7)	
MNA				
At risk of malnutrition	60 (28.0)	48 (31.2)	12 (20.0)	0.102
Normal nutritional status	154 (72.0)	106 (68.8)	48 (80.0)	
BI				
Partially dependent	14 (6.5)	11 (7.1)	3 (5.0)	0.739
Minimally dependent	149 (69.6)	108 (70.1)	41 (68.3)	
Independent	51 (23.8)	35 (22.7)	16 (26.7)	
MMSE				
Cognitive impairment	84 (39.3)	69 (44.8)	15 (25.0)	0.008
Without cognitive impairment	130 (60.7)	85 (55.2)	45 (75.0)	

W/S/D, widow/separated/divorced; WC, waist circumference; BMI, body mass index; MNA, mini nutritional assessment; BI, Barthel index; MMSE, mini mental state examination. In brackets are the percentages: statistical comparisons between genders.

**Table 2:** Age effects on nutritional status, nutritional risk, self-dependence in activities of daily living, and cognitive status.

Variables	Age Groups			*p*-Value
	65–76	77–86	87–99	
MNA				
At risk of malnutrition	10 (16.7)	33 (55.0)	17 (28.3)	0.599
Normal nutritional status	35 (22.7)	81 (52.6)	38 (24.7)	
BMI				
Underweight	1 (6.3)	8 (50.0)	7 (43.8)	0.225
Normal	20 (27.0)	37 (50.0)	17 (23.0)	
Overweight	24 (19.4)	69 (55.6)	31 (25.0)	
WC				
Normal	29 (22.0)	64 (48.5)	39 (29.5)	0.165
High risk	16 (19.5)	50 (61.0)	16 (19.5)	
BI				
Partially dependent	3 (21.4)	8 (57.1)	3 (21.4)	0.943
Minimally dependent	10 (24.4)	22 (53.7)	9 (22.0)	
Independent	32 (20.1)	84 (52.8)	43 (27.0)	
MMSE				
Cognitive impairment	20 (23.8)	43 (51.2)	21 (25.0)	0.723
Without cognitive impairment	25 (19.2)	71 (54.6)	34 (26.2)	

MNA, mini nutritional assessment; BMI, body mass index; WC, waist circumference; BI, Barthel index; MMSE, mini mental state examination.

**Table 3:** Participants’ nutritional risk and functioning based on their BMI categorisation.

Variables	BMI			*p*-Value
	Underweight	Normal	Overweight	
MNA	21.8 ± 2.5 ^a^	25.0 ± 2.0 ^b^	25.3 ± 2.2 ^b^	<0.001
BI	74.7 ± 18.5 ^a^	84.8 ± 15.7 ^b^	86.0 ± 14.4 ^b^	0.021
MMSE	19.0 ± 5.6	21.1 ± 4.9	21.1 ± 5.3	0.295

MNA, mini nutritional assessment score; BI, Barthel index; MMSE, mini mental state examination. ^a,b^ Values in rows with different superscripts are significantly different.

**Table 4:** Participants’ nutritional status and functioning based on their MNA categorisation.

Variables	MNA		*p*-Value
	At Risk	Well Nourished	
BMI (kg/m^2^)	27.1 ± 5.6	28.8 ± 4.5	0.018
BI	77.0 ± 17.8	87.7 ± 13.2	<0.001
MMSE	19.3 ± 5.6	21.6 ± 4.9	0.003

MNA: mini nutritional assessment; BMI: body mass index; BI: Barthel index; MMSE: mini mental state examination; WC: waist circumference.

**Table 5:** Correlation between nutritional status, nutritional risk, and functional and mental disability.

Variables	BMI		WC		MMSE		BI	
MNA	0.28	(<0.001)	0.13	(0.059)	0.30	(<0.001)	0.40	(<0.001)
BMI			0.72	(0.715)	0.08	(0.239)	0.09	(0.169)
WC					0.01	(0.899)	−0.04	(0.572)
MMSE							0.35	(<0.001)

MNA: mini nutritional assessment; BMI: body mass index; BI: Barthel index; MMSE: mini mental state examination; WC: waist circumference. (In brackets are the *p*-values).

## Data Availability

The data presented in this study are available on request from the corresponding author.
